# Validation of the FROM-16 in family members of patients receiving advanced therapy medicinal product (ATMP)

**DOI:** 10.1007/s11136-024-03880-0

**Published:** 2025-01-25

**Authors:** Charles D. Brilliant, Andrew Y. Finlay, Sam M. Salek, Rubina Shah, Emily Bacon, Hamish Laing

**Affiliations:** 1 Value-based Health & Care Academy, School of Management, Swansea University, Swansea, SA1 8EN UK; 2 Division of Infection and Immunity, Cardiff University School of Medicine, Cardiff, CF14 4XN UK; 3 Public Health & Patient Safety Research Group, School of Life & Medical Sciences, University of Hertfordshire, Hatfield, AL10 9AB UK; 4 Institute of Medicines Development, Paddock End House, Bucks, SL9 8BL UK

**Keywords:** FROM-16, Advanced Therapy Medicinal products, Quality of life

## Abstract

**Purpose:**

Outcome-based pricing models which consider domains of value not previously considered in healthcare, such as societal outcomes, are of increasing interest for healthcare systems. Societal outcomes can include family-reported outcome measures (FROMs), which measure the impact of disease upon the patient’s family members. The FROM-16 is a generic and easy-to-use family quality of life tool, but it has never been used in the context of patients undergoing advanced therapy medicinal product (ATMP) treatment. The use of potentially curative ATMPs is limited due their high cost and the low number of eligible patients. Using the FROM-16 to collect the impact on family of disease and treatment in ATMP patients may demonstrate additional value created by an ATMP intervention and strengthen the case for its use.

**Methods:**

This feasibility study aimed to test the validity of the FROM-16 in family members of ATMP patients as a prelude for its use in ATMP value estimation. Patients and family members (*n* = 24) were recruited from ATMP treatment centres in England and Wales. Family members completed the FROM-16 and were invited to a short debriefing interview.

**Results:**

The FROM-16 showed high validity demonstrated by strong internal consistency (Cronbach’s alpha = 0.917) and intraclass correlation (0.803, 95%). Interviews identified that whilst the FROM-16 covered most areas of quality-of-life impact experienced by the participants, some explained that they also experienced other impacts upon their personal health and future outlook.

**Conclusion:**

This feasibility study provides evidence that the FROM-16 could be used as part of a structured systematic approach to measure family quality of life impact in ATMP patients.

**Supplementary Information:**

The online version contains supplementary material available at 10.1007/s11136-024-03880-0.

## Introduction

### Wider Impact & Value Based Health Care

Family members who live with or care for a relative with a chronic disease may suffer equally or sometimes even more than the patient themselves [1–9]. They may experience stress, anxiety, depression, fear, anger and helplessness, which may exacerbate any existing stressors, pressures or illnesses, regardless of whether they assume any caring responsibilities. Family members may also experience significant social impacts, such as social isolation and restricted ability to work or study [10, 11]. These impacts form part of the “Greater Patient” [8], the needs of which must be understood fully if they are to receive appropriate support.

Value, defined in healthcare as outcomes that matter divided by the cost of achieving those outcomes [12, 13], can be considered in four domains: Personal, Allocative, Technical and Societal [14]. Societal value recognises that improved outcomes for a patient may create additional value beyond that achieved for the patient themselves. This could support the use of high cost and resource-intensive interventions if the societal value created is sufficient; for example through reduced impact upon family member quality of life and productivity [14].

### Advanced Therapy Medicinal products

Advanced therapy medicinal products (ATMPs) are a heterogenous class of therapeutics originating from modified human biological materials, comprising gene therapies, stem cell therapies, tissue engineered therapies and combined advanced therapies [15–17]. ATMPs offer potentially curative treatment opportunities for rare conditions and have been successful in genetic and metabolic disease, degenerative neurological disease, oncology, haematology, cardiology and orthopaedics [18]. They can have a significant positive impact on the quality of life of the patient and their family [19].

Widespread deployment of ATMPs is limited, partially due to healthcare payers being unwilling to take on the very high associated costs. Considering the health, social and economic “*value created”* by an ATMP may justify these costs, and doing so can inform decision-making at a policy level [20]. However, current approaches of some health technology assessment (HTA) agencies do not consider wider socioeconomic benefits as strongly as disease burden, or cost-effectiveness of providing the intervention when assessing the value of an intervention [21, 22]. It may be necessary to broaden the definition of value in the context of ATMPs to be able to include components of value not previously considered, such as societal outcomes [22]. Value-based pricing models, where consideration of value requires demonstration of positive or desired outcomes beyond cost-effectiveness, are expected to become more prevalent [23].

Value-, or outcome-based pricing agreements require reliable and validated tools for collecting patient-reported outcome measures (PROMs) and family-reported outcome measures (FROMs). Currently, FROMs are not routinely collected and collection of PROMs in clinical practice is still limited, although it is becoming more widespread. The NHS is expanding its requirement to collect PROMs and guidelines have been published to conduct pilot and feasibility PROM studies for new PROM tools [24]. FROMs are designed to collect evidence of quality-of-life impact in family members of patients, however, despite the evidence of wider impact of disease there is no current requirement to measure this impact. In the context of ATMPs, which can have a significant positive impact on both patients and their family members, and can create a net societal benefit [19], collection of FROMs may be crucial in demonstrating components of the wider family and societal impacts of ATMP treatments.

### Family-reported outcome Measure-16

The Family-Reported Outcome Measure-16 (FROM-16) is a generic quality of life questionnaire designed to measure disease impact on family members of patients across all medical specialities. It consists of 16 short items across two domains: Emotional (comprising six items, maximum score of 12) and Personal and Social Life (comprising ten items, maximum score of 20) and takes two minutes to complete [25]. The key themes include emotional impact (feeling of being worried, sad, frustrated, angry and difficulty in sharing thoughts and caring) and personal and social impact (impact on time for self, travel, eating habits, family activities, sex life, holidays, work and study, family relationships, family expenses, and sleep).

Initial development resulted from semi-structured interviews with 133 family members of patients across 26 medical specialities, exploring in depth the impact of a relative’s health condition on family members. A preliminary 31-item measure developed from the content of the interviews with family members was reduced to a 16-item measure following Rasch analysis and factor analysis (*n* = 240). Content validity was assessed using qualitative and quantitative data from expert panels involving clinicians and family members. The initial validation of FROM-16 demonstrated high internal consistency (*n* = 120, Cronbach’s α = 0.91), and high reproducibility (*n* = 51, ICC = 0.93). Construct validity was proven through the correlation between the FROM-16 and the WHOQOL-BREF total scores (*n* = 119, *r*=-0.55, *p* < 0.001), and the correlation between the FROM-16 and the patient’s overall health score (*n* = 120, *r*=-0.51,*p* < 0.001)^25^. In recent years, FROM-16 has undergone extensive further validation. The interpretation of scores is described using validated score meaning bands [26]. Responsiveness to change has been established [27] and the Minimal Important Change (MIC) value of FROM-16 has been estimated as a score change of four points [27]. Furthermore, the FROM-16 has been mapped to EQ-5D-3 L [28] for the potential use of inclusion of family impact of disease in health economic analysis. The FROM-16 has been validated across 26 medical specialities [29] and in additional conditions including urological, cancer, COVID-19 and myalgic encephalitis as well as in multiple languages and cultures [30–36]. Although the FROM-16 is a reliable tool for measuring family impact across a wide range of medical disciplines, it has not yet been applied to a population of ATMP patients and their families, which have some unusual features such as very high treatment costs, often for inherited conditions with others in the family affected but not offered ATMP treatment.

### Study aims

This study aimed to validate the use of the FROM-16 as a tool for measuring family impact as part of value estimation in ATMP patients by recruiting family members of patients attending ATMP-provider clinics in the Midlands & Wales Advanced Therapy Treatment Centre (MW-ATTC).

## Methods

### Recruitment

#### Ethical approval

was obtained from the Yorkshire & The Humber -Sheffield Research Ethics Committee (REC no: 21/YH/0228). Recruitment of participants took place in four ATMP treatment centres within the extended MW-ATTC network: Cardiff & Vale University Hospital, University Hospital of Leicester, University Hospital of Birmingham & University Hospital of Bristol & Weston. Through note review, local research nurses identified potential participants, who were approached directly by their usual clinical team, who provided the study information and obtained informed consent. Participants were afforded the opportunity to provide consent and participate fully remotely, if they wished to.

### Patients

Patients of any age were eligible if they could read English or Welsh (if age-appropriate), attending an ATMP treatment centre with a family member and were being considered for, or had recently received, ATMP therapy for any diagnosed condition. Patients were not active participants in the research.

Patients were excluded if they were not eligible for ATMP therapy, or if they lacked capacity to give informed consent, or no consent was given by a parent/guardian. Family members were excluded if they were under 18 years of age, not considered family by the patient, did not or could not provide informed consent to participate, or if they lacked capacity to complete the questionnaire and/or interview.

### Family members

Family members were eligible if they were aged 18 years or older, could read English or Welsh, had capacity to give written informed consent and were considered by the patient to be the family member most affected by their condition.

### Data collection

#### Questionnaires

The questionnaires used included the FROM-16 and a global health score (GHS) question as a measure of the patient’s overall health. The GHS was a single question with responses measured on a Likert scale (“Thinking of your affected family member, on a scale of 0–10 (worst to best), how severe do you consider that their disease is now?”).

Basic demographic information of the patient and family member, as well the ATMP administered or proposed and the patients’ diagnosis, were collected.

#### Interviews

Family members were also invited to a two-stage, semi-structured tele-interview. Ten interviews were carried out, with one additional participant providing free-text on the reverse side of their returned FROM-16 questionnaire. Interviews were carried out in two-stages: (1) cognitive debriefing; and (2) discussion of impact/change. The cognitive debriefing used the same validated question-set applied to assess the content validity of the FROM-16 in other disease populations: [25]

1. “Is the questionnaire easy to complete?”;
2. “Are the response options straightforward?”;
3. “Are the instructions and statements clear?”;
4. “Do the questions cover all areas of your life which have been affected?”;

with one additional question:

5) “Did you feel that any of the questions in the FROM-16 were not applicable to your situation?”.

During the second stage of the interview, participants were encouraged to provide additional context to their FROM-16 responses. The interviewer used the Most Significant Change framework to guide participants to describe the most important changes to their life without pre-defined indicators [37].

Interviews were recorded and transcribed verbatim. Interview transcripts and any participant generated free text was analysed using NVivo 11 to identify key themes [38].

### Data Analysis

Content validity of the FROM-16 was assessed through the cognitive debriefing interviews by measuring the intraclass correlation of responses to the questions.

Themes were identified from the interviews and free-text using a combined inductive-deductive approach. Interview responses were initially coded with respect to the FROM-16 items with sub-codes assigned as necessary to contextualise the FROM-16 responses by showing distinctions between levels of impact (e.g. item 16 “my sleep is affected” was sub-coded into item 16.1 “negative affect on sleep” and item 16.2 “positive affect on sleep”). Interview responses not relating directly to any FROM-16 items were coded inductively.

Statistical analysis was performed using the R statistical package [39]. The irr [40] and ltm [41] packages were used to assess intraclass correlation using a two-way ANOVA model and internal consistency using Cronbach’s alpha, respectively. Dimension reduction and confirmatory factor analysis was performed using SPSS and AMOS, respectively [42].

## Results

### Recruitment

**Fig. 1:**
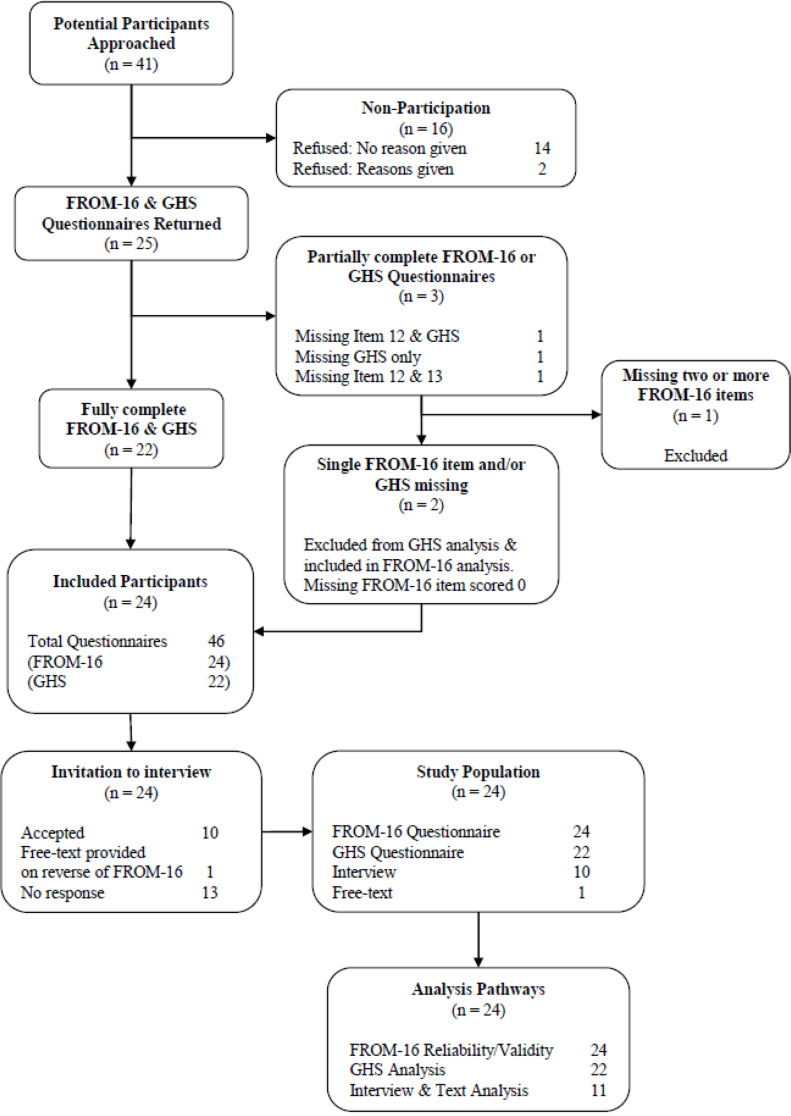
STROBE diagram of study recruitment and analysis pathway

Forty-one patient and family member pairs were invited to participate. Twenty-five returned completed consent forms and the questionnaires. Of these, 24 returned fully complete FROM-16 and 23 returned fully complete GHS. Participants with missing data were excluded if there was more than one missing item (Fig. 1). Ten participants agreed to take part in the interview, with one providing free-text on the reverse side of the questionnaire. Two non-participants gave a reason for not wishing to take part (Fig. 1). One, who was a family member, “*did not wish to delve into their feelings*” on the matter. The other, who was a patient, had recently received an ATMP and felt “*too overwhelmed to engage with the study*”.

### Demographics

The majority of participants were family members of adult patients (92%) and two were parents of paediatric patients. Of the adult patients, 50% were aged 61 years or greater. Participants were predominately in long-term partnerships (married/civil partner/etc.). The gender of the patients was evenly distributed between male and female, but 66.67% of the family members were female. The ethnic group of the participants was predominantly White British (Supp. Table 1).

### Diagnoses

Both paediatric patients were diagnosed with Type 1 Spinal Muscular Atrophy (SMA) and received Zolgensma™ (Onasemnogeneabeparvovec, Novartis Gene Therapies) (Supp. Table 2). The majority of adult patients (19/24) were diagnosed with a malignancy although the site, pathology and subsequent ATMP administered varied. Patients with malignancy predominantly were prescribed chimeric antigen receptor therapy (CAR-T). All but one patient recruited had already received an ATMP, with the remaining participant being prepared to receive an ATMP. The mean duration of the condition from initial diagnosis to treatment being received was 39.6 months (SD = 33 months).

## Results

### FROM-16 and GHS scores

Summarised FROM-16 responses (Table 1) showed that item 2 (“I feel angry”) had the lowest mean score (0.63) and that item 1 (“I feel worried”) had the highest mean score of 1.54. The participant who had a total score of 1 reported their only impact as being “A little worried”.

The mean total FROM-16 score was 15.7, SD 8.51, (Median = 14) (Table 1). The FROM-16 validated score band of “9–16” means “a moderate effect on family member” [43]. The number of family members whose scores fell into the various bands are as follows: FROM-16 score 0–1, meaning “no effect on the quality of life of family member”, one family member; 2–8, meaning “small effect”, four members; 9–16, meaning “moderate effect”, eight members; 17–25, meaning “very large effect”, seven members; 26–32, meaning “extremely large effect”, four members.

The GHS showed a mean score of 6.18, SD 2.4 (Median = 6.5) (Table 1). There was no linear relationship between GHS and total FROM-16 scores (Spearman’s Rho 0.02), and no relationship between any individual FROM-16 item score and GHS scores. The item with strongest relationship with the GHS was item 4 (“I feel frustrated”), which was moderately weak (Spearman’s Rho − 0.338).

**Table 1:** FROM-16 items scores and GHS showing the measures of central tendency*, with comparison to previously published FROM-16 total and individual domain scores. #; Data is not available

Item		Mean	Median	Mode	Standard Deviation					
Domain 1	Item 1 I feel worried	1.54	2	2	0.66					
	Item 2 I feel angry	0.63	1	0	0.65					
	Item 3 I feel sad	1.25	1	1	0.68					
	Item 4 I feel frustrated	1.04	1	1	0.75					
	Item 5 It is difficult to find someone to talk to about my thoughts	0.83	1	0	0.82					
	Item 6 Caring for my family member is difficult	0.71	0.5	0	0.81					
Domain 2	Item 7 It is hard to find time for myself	0.83	1	0	0.87					
	Item 8 My everyday travel is affected	0.71	0	0	0.91					
	Item 9 My eating habits are affected	0.71	0	0	0.91					
	Item 10 My family activities are affected	1.29	1.5	2	0.81					
	Item 11 I experience problems with going on holiday	1.42	1.5	2	0.65					
	Item 12 My sex life is affected	1.04	1	2	0.95					
	Item 13 My work or study is affected	0.96	1	0	0.95					
	Item 14 My relationships with other family members are affected	0.71	1	0	0.75					
	Item 15 My family expenses are increased	0.88	1	1	0.74					
	Item 16 My sleep is affected	1.17	1	1	0.76					
GHS		6.18	6.5	8	2.40					
Mean Totals (current study)						Mean Scores from previous studies (references)				
						^25^	^27^	^28^	^30^	^40^
**Domain 1 – Emotional**		6.00	5	4	2.95	5.6	4.7	#	#	#
**Domain 2 – Personal & Social Life**		9.71	8.5	5	6.13	6.7	7.1	#	#	#
**Total FROM-16 Score**		15.71	14	10	8.51	12.3	11.8	16.8	10.7	15.02

*FROM-16 questionnaire, reproduced by permission

©M.S.Salek, A.Y.Finlay, M.K.A.Basra, C.J.Golics, May 2012

### FROM-16 validity and reliability

All participants reported in the cognitive debriefing interview that they found the questionnaire easy to complete (Q1), with straightforward response options (Q2) and clear instructions (Q3). In response to Q4, 50% of interviewees felt that all impacted areas of their life had been appropriately covered by the FROM-16, but three participants stated that they felt their health had been negatively impacted by their family member’s illness but could not record this appropriately on the FROM-16. Of the remaining interviewees (*n* = 2) one discussed how the FROM-16 does not address their future outlook, and the other participants referenced the impact of the COVID pandemic on their life. Interestingly, during the later stages of the interviews all interviewees went on to describe areas of impact beyond the FROM-16 items. Responses to Q5 showed that 70% of participants felt all items on the FROM-16 were relevant to their situation. The reasons given by the participants who considered that certain FROM-16 items were not relevant were primarily related to those items not being impacted for that participant. A common theme was that item 13 (My work or study is affected) was not relevant as the participant had already ceased work for other reasons (COVID-19, or retirement). Content validity was shown to be high, confirmed by an intraclass correlation of 0.882 for “Yes/No” coded responses to the cognitive debriefing interview questions.

The internal consistency reliability of the FROM-16 was shown to be excellent, demonstrated by Cronbach’s alpha of 0.917, (95% CI 0.856: 0.948). This agrees with Golics et al. [25] who also reported excellent internal consistency of the FROM-16. The FROM-16 also showed good-to-excellent intraclass correlation (0.803, 95% CI [0.631 : 0.918]) using a two-way average score model.

Comparing the FROM-16 responses from this study to previously published FROM-16 validation and development studies showed comparable mean scores across both the emotional and personal and social life domains, and the total score (Table 1). The impacts reported by participants in both domains in the current study are slightly higher than those reported in previous studies, although this may be expected due to the high severity and presumed high impact of the patients’ conditions.

### Confirmatory factor analysis

**Fig. 2:**
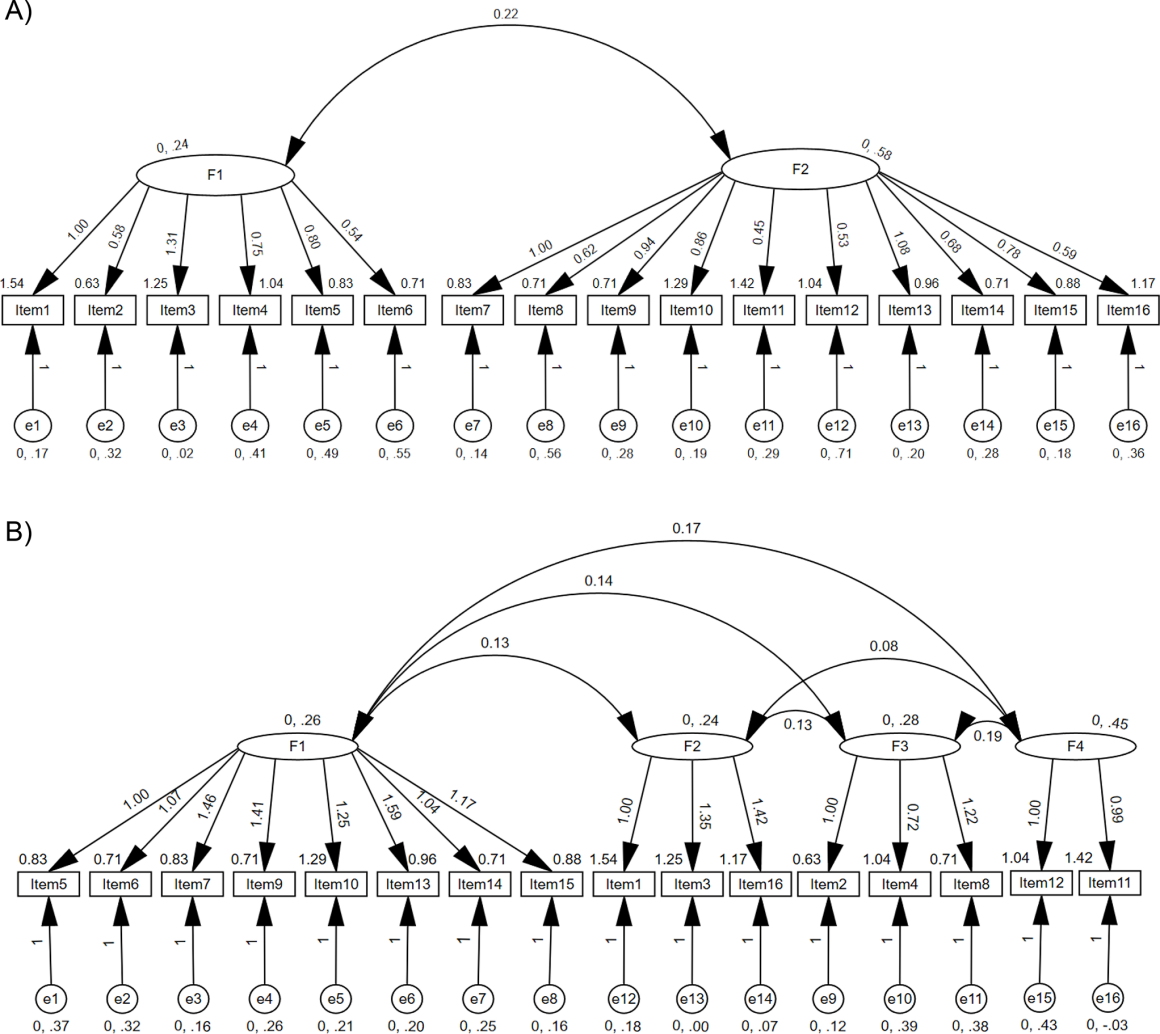
**A**) Two-factor, and **B**) four-factor model structures, with item and error estimates shown

Confirmatory factor analysis examining a two-factor model (Fig. 2) with items 1 to 6 in factor 1 and items 7–16 in factor 2, showed only a moderate fit (Comparative Fit Index (CFI): 0.542) and a Root Mean Square Error of Approximation (RMSEA) of 0.237, above the recommended threshold of 0.06 [31]. Dimension reduction identified four components with Eigen values in excess of 1; a four-factor model showed a moderately better fit, with a CFI of 0.783 but with a corresponding high RMSEA (0.167). The moderately strong fit shown by the four-factor model may be a result of over-fitting due to the small sample size.

### Interview thematic analysis

Deductive coding into themes associated with the individual FROM-16 items showed that 15 out of 16 items were mentioned by participants at least once during the interviews (Supp. Table 3). The item not mentioned was item 5 (“It is difficult to find someone to talk to about my thoughts”), which had a mode score of 0, and a mean of 0.83 (Table 1), indicating that this component of the participants’ lives had not been greatly impacted upon or changed in a majority of participants. It is worth noting however, that of the interviewed participants, 60% reported a score of 0 for item 5, as opposed to ~ 40% of all participants. As the majority of interviewed participants reported zero impact, this may explain why they did not feel it was important to discuss. Six items were further coded into sub-themes to distinguish between positive and negative references made by the participants, for example, item 2 “I feel angry”, was further sub-coded in to ‘2.1 Angry’ and ‘2.2 Not angry’ (Supp. Table 3).

#### Positive and negative impacts

All interviewees discussed positive and negative impacts in their social life. Participants described feeling supported by their friends and communities as a direct result of their relative’s disease, feeling closer to them as a result (Table 2). For example, one participant expressed that another relative with a medical background *“was very helpful when my husband was so ill*,* she was talking to the consultants*,* and she knew the right questions. And it took a lot of the pressure off of me”*. Conversely, significant negative impacts were also described, mostly due to feeling isolated from friends and social activities, or particularly in the case of a parent of a paediatric patient, feelings of stigmatisation or “otherness” when attending baby support groups (Table 3). One participant shared particular feelings of isolation because, due to shielding, *“[my husband] sees more people than me at the hospital*,* whereas I just have to wait in the car”.* The impact of not spending as much quality time with their family member was also noted: one participant stated that she was used to *“doing everything”* with her husband, with his disease now preventing them from doing so. Participants also described increased hospital admissions and being unable to see their relative for weeks, expressing that “*we’ve never been apart for that long*”: these participants stated heightened feelings of loneliness and isolation as a result.

Participants also discussed theeffects on their relationships with other family members, however this was not necessarily a negative impact. Some participants reported positive impacts, such as feeling closer to their relative, or other family members becoming more involved in childcare (Table 4). Alternatively, the negative impact on relationships with family stemmed from missing out on family events, or even a lack of understanding of the disease from family members (Table 5). This variable nature of the impact may explain the relatively low mean impact reported by the participants for item 14 (Table 1).

**Table 2:** Positive impacts of the patient’s disease on their Social Life

Item/Code	Example quotes
**Positive impact on social life**	Well, the good thing that’s come out of it is how I feel so fortunate and blessed with my family and my friends, and my church
	I must admit, I’ve got good friends, and everybody used to ring me up to find out how I was and how [NAME REDACTED] was, you know, family as well. Always used to ring me. And if I needed to talk, you know, I could always ring them, which was nice, and sometimes I needed that.

**Table 3:** Negative impacts of the patient’s disease on their Social Life

Item/Code	Example quotes
Negative impact on social life	Like I haven’t been into church and we were regular attenders for two years
	Oh, for two years he hasn’t been out except has to keep his hospital appointments… we’ve always done more-or-less everything together
	He wasn’t up to doing a lot, you know, so our social lives sort of came to a stop for a while because he just didn’t feel like he wanted to go out or do anything. You know, he was tired, you know, lethargic. It made a lot of difference to our social life. And we played skittles as well. You know, we’re in the skittles team. So he couldn’t do that very often because he didn’t feel up to it.
	Like I want to take her to like social baby groups and things. But then I got the other side of me with like the stigma. Because she’s on, like, an NG-tube, and she’d be the only one in the group like that. They’d be wondering, like, why is she still holding her at that age? You know, I got all that against me.
	Can’t even…. because they’re low-immune and all the rest of it, we can’t even contemplate going out

**Table 4:** Positive impacts of the patient’s disease on their relationships

Item/Code	Example quotes
Positive impact on relationships	The positive things are obviously, you know, that we talk a lot and my husband does a blog about his condition. So it’s sort of, the wider family are much closer now. People are talking and phoning each other and things like that. So it’s actually sort of brought the family closer together in that respect
	And I suppose, you know, as a positive, we sort of appreciate each other’s company more. We don’t take each other for granted like we did.
	So, you know, we’d been sort of this very much the two of us, I suppose. More so than we were before. You know, we were doing our own things and whatever, which was nice. But I suppose we tend to do more together now.
	And I suppose we were fortunate enough that we had family that rallied around us to help us in all sorts of different ways. So again, I suppose my relationship with other family members are affected in one sense. Yes, not necessarily… Positively

**Table 5:** Negative impacts of the patient’s disease on their relationships

Item/Code	Example quotes
Negative impact on relationships	I feel like we’re missing out on like the rest of the family’s, things going on. Like she’s got cousins who only live like 30 min away, but like, she hasn’t really built a relationship with them, they’re all around the same age
	So that impacted our lives greatly as we were only seeing like two sets of grandparents. Now, that was unhealthy for me personally
	And we had the opportunity to go on holiday with our family from New Zealand, to Thailand, but we couldn’t because [NAME REDACTED] had to be back. And it was [NAME REDACTED] sister’s 70th. And because we had to be back for a day where she had to be at UCL for an interview. And obviously they couldn’t understand why we couldn’t go
	My relationship with family members means that it’s quite often affected because […]. If I say I’m doing something with a family member, it’s always on the proviso “has [NAME REDACTED] got a hospital appointment?”
	Yes, the effects are I don’t get to spend as much time with my daughter, who is ten years old. And so, my relationship with her is affected by what’s going on

#### Personal importance of impact

A theme emerged around the importance of an impact to the family member, and how the importance ascribed to an impact is dependent on the family member’s or patient’s current circumstances. For example, a participant may report an impact on the FROM-16 on their ability to go on holiday, however in the interview they discussed that they do not consider this impact to be important to them (Table 4).

The majority of participants (80%) interviewed used phrases to underplay or diminish the impact on certain areas of their life, such as “*but I got used to it*”, “*we muddled through it*”, or “*it’s not a problem*”. These phrases were used across multiple domains, including sex life, impact upon work, relationships with other family members, and impacts upon social life and activities. This however contrasted with participants expressing that *“life used to be easier”* but it had now “*deteriorated*”. Participants described developing time management strategies and trying to maintain normality in their life by not sharing their experiences with other people. These statements indicate that the family members experienced difficulties, perhaps phrasing these difficulties as such due to feelings of guilt or self-embarrassment around finding caring for a loved one difficult.

Moreover, participants downplaying personal impact is perhaps due to participants not wanting to seem burdened by their family member’s disease. As demonstrated in Table 6, it further illustrates a participant refocus on ‘what is important’, that being the health and quality of life of their family member. Additionally, it demonstrates a potential coping mechanism for dealing with the impact (Table 7).

**Table 6:** Downplaying impact

Item/Code	Example quotes
Downplaying impact	…at any given time, any one of those could be at the forefront of being a particular issue. I mean, each of these has been an issue at various different times. I mean, that the whole thing about going on holiday. Yes. It’s massively stopped me going on holiday. But is it an important aspect to me? Absolutely not. I’m not worried about not going on holiday and family activities for.
	And I think the great positive thing is, is that it’s really made us reflect on our current life and made us more grateful for the things that we have
	And I don’t think we’ve had more than 2 or 3 weeks, other than post-op, when he hasn’t had scans, tests, hospital appointments, which of course, we have prioritised and it’s not a problem to prioritise
	I didn’t mind finishing work at all apart from the financials
	You asked about sex life, not that that’s irrelevant but there’s no way we could have considered it. I mean, I think about the surgery and how ill he’s been. A cuddle and hug means the world, but that’s about it. Um, I struggle to see anybody going through what we’ve been through that could even contemplate anything like that. To be honest, because it becomes irrelevant almost.
	I’d say she’s till the same child that I would have had anyway. Just with a few more bits added on. I’m not treating her any different to any other child or anything

**Table 7:** Coping strategies

Item/Code	Example quotes
Coping strategies	You know, because I couldn’t go shopping so I’ve been stuck in with him for two years, but I’ve got used to it!
	You do it and you get through it because you’re strong
	But we’re very lucky that we’re a strong unit and we’ve muddled through.
	No, I’ve always taken my frustration out on the garden
	So it was more about trying to manage our time and what we were doing.
	But you know it’s urm, you just take every day as it comes, you know
	We can’t always be spontaneous about the things we do because [NAME REDACTED] is always having to go for appointments or treatments or tests or something. But we think it’s a small price to pay for the treatment, ongoing care and treatment that she’s getting now.
	I think that there has to be normality in life. And I think the fewer people that actually do know, the more normal your life can be, apart from those ones that I truly trust.

#### Future Outlook

Some participants also reported that a significant impact on their lives was an inability to plan for the future due to uncertainty around prognosis or the treatment (Table 8).

**Table 8:** Impact on future

Item/Code	Example quotes
Impact on future	Yeah, I think it’s relevant because I think it’s relevant to assess somebody’s mental health as well, that they feel that they are able to look forward to something. Because when you are going through something like this, that was truly horrendous, and impacts on every aspect of your life. I think you cannot look forward, you can only cope with that day, that week.
	We used to do a lot of long term planning. And I suppose it sort of made me realize, you know, we can’t plan too far ahead
	I mean, we’re hoping he’ll have a nice long spell of good health, but I think it stopped us from planning way ahead just like we used to
	if you wanted me to sum up, I think the worse thing is, you know, not being able to plan long term. I think that’s how it’s affected both of us the worst of everything.

**Table 9:** Impact on personal health

Item/Code	Example quotes
Impact on personal health	But it doesn’t sort of relate to the fact that, myself or someone in my situation might also have health issues and how that then the interaction between two different health issues.
	I suppose you could say my health perhaps isn’t involved because I had I’ve had awful toothache, which, because of my husband’s treatment, him being vulnerable and COVID-19. I put up with toothache for about three months.
	At times it was very difficult, especially when I was suffering my own ill-health (haemolytic anaemia)

Participants reported “*unease*” about the future or changing of their outlook towards a shorter-term perspective. In fact, when asked “Do the questions cover all areas of your life which have been affected?” one participant responded that a component related to future outlook was missing from the FROM-16:

…probably, apart from how do I feel moving forward?

The ability to plan, or look forward to events in the future, was highlighted as a major indicator of mental health and the loss of that due to the patient’s disease was reported to have a significant negative impact upon a family members mental health (Table 8). Participants further stated how they were more inclined to live in the moment and not *“take things for granted”.* The various impacts of continually prioritising their relative’s disease over their own life was also evident across all areas of participants’ lives, including inability to work due to caring responsibilities (financial), changes in eating habits (physical), being unable to meet up with friends (social) and having no time for themselves (mental). Interestingly, impacts upon physical health were reported despite this not being explicitly measured on the FROM-16. Participants identified this a missing item in response to “*Do the questions cover all areas of your life which have been affected?”* and discussed how they had neglected their own health in favour of their relatives (Table 9).

## Discussion

Family members caring for relatives with severe disease can experience significant impacts, which are often unrecorded. Measuring these can help to understand the wider impact that a patient’s disease can have on their family and wider society, and therefore can help to demonstrate potential greater value of treatment. The FROM-16 is a validated tool for collecting family-reported outcome measures across a broad range of medical disciplines but has not been used previously to measure the unique impacts in the heterogenous group of patients receiving ATMPs and their families. Therefore, we aimed to validate the FROM-16 in this population.

When applied to family members of ATMP patients, the FROM-16 showed high reliability, through excellent internal consistency (Cronbach’s alpha = 0.917) and intraclass correlation (0.803). The validation of FROM-16 was carried out on a mixture of family members of adults and of children [25, 26] but there has been no separate validation in adult family members of children. However, in the original psychometric development and testing of the FROM-16, family members of paediatric patients were also included [25, 26]. The use of ATMPs is usually confined to adult patients, explaining the small number of paediatric patients in this study. Confirmatory factor analysis showed only moderate fit to a two-factor model with a high RMSEA, only slightly improved in a four-factor model, which also showed a high RMSEA. Two previous studies of CFA [44, 45] demonstrated a better fit for the two factor model than the current study, confirming the original findings of Golics et al. [25]. A larger study may allow the generation of a more meaningful model.

Cognitive debriefing demonstrated high content validity. All participants reported that the FROM-16 was easy to complete, and non-burdensome. Most participants felt the FROM-16 addressed all areas of their life that had been impacted: One participant described the FROM-16 as “quite generic” and commented that it could be applicable even to a person who had just had a baby. Others also considered some items not to be relevant to their specific experiences. These comments reflect a specific design principle of the FROM-16, which is for it to be applicable to families of both adult and paediatric patients with a wide range of different conditions and illnesses and family circumstances as well as lived experiences. Therefore, we do not consider these views to reflect a limitation of the FROM-16 but rather a positive attribute: quality of life impact can be variable and is dependent on the current circumstances of the individual. The FROM-16 reflects the concerns reported by 130 family members/partners of patients from a wide range of medical specialties. It is therefore encouraging that a FROM that was conceptualised as a generic instrument was perceived as one.

It is of note that 50% of interviewees, when initially asked in the cognitive debriefing interview (Q4) stated that the FROM-16 covers all areas of their life that have been impacted by their family member’s illness. However, during the later stages of the interview process, where participants were afforded more freedom to discuss their feelings and perceptions of impact/change to their life, all (100%) of interviewees reported themes that were beyond the scope of the FROM-16. This indicates that whilst the FROM-16 is a viable, generic tool for assessing impact this population, and will generally cover the areas expected by the end-user, some additional questioning, or listening service by the healthcare professional administering the FROM-16 could be beneficial for identifying additional areas of impact. Some of these impacted areas are discussed below.

The majority of interviewees attempted, at least once, to downplay the impacts they felt from their family member’s illness. Reasons for this were varied, such as not ascribing importance to the impacted area, or feeling as if the impact they feel was overshadowed by more important issues. Some participants also used coping strategies to lessen their perceived impact, which require additional mental or physical efforts from the family carer. We consider this to indicate a hidden or silent impact that could lead to ‘burnout’, and even depression in the family carer. This is a potential limitation of the FROM-16, which does not ask the family member to consider how important items are to them, regardless of the scale of the impact. Therefore, if using the FROM-16 to identify areas of unmet need in family carers, especially if the goal is to direct the family carer to appropriate support, consideration must be given to the importance that the family member ascribes to the impacted area of their life. This could be achieved through the routine use of a structured systematic assessment of family carers, including use of the FROM-16, to help detect hidden or silent impacts before they progress to greater future impacts.

This is highlighted additionally by participants reporting both positive and negative impacts across multiple areas of their lives (Tables 2 and 3). The FROM-16 does not have a utility for qualifying impacts experienced and may encourage a user to consider negative impacts only. Family members must have an opportunity to discuss the nature of any impacts they experience during any routine monitoring.

Participants also reported neglecting their own health or struggling more with their own health issues as a result of their relative’s illness, which is also not measured by the FROM-16 (Table 4). Any routine assessment of family impact in ATMP patients should include a measure for tracking perceived impacts upon physical health of the family members, to bring this impact to light, and prevent it being part of the hidden wider impacts of disease. This will facilitate the allocation of support/resources to family members who require assistance and may prevent worsening impacts in the future.

Participants discussed that long-term planning, or the ability to “*look to the future*”, which they reported as having been given back to them as a result of the ATMP, also was not captured by the FROM-16 (Table 8). This suggests that when assessing impact in families of ATMP patients, an additional dimension or measure examining their perception on future outlook may be important.

This study has added to the pool of FROM-16 validation studies, with comparable scores to previous validation and development studies, however it is of interest to note that the scores reported in the current study are slightly higher (Table 1). The majority of patients recruited to this study had already received an ATMP treatment, so a reduced impact might be expected. However, family members reported ongoing impacts such as continuing to attend hospital appointments and uncertainty about the long-term treatment success as well as feelings of relief and being able to look to the future again as a direct result of the ATMP. This may explain the slightly higher total and domain scores shown in the current study. This suggests that the FROM-16 may be equally valuable in longer term monitoring; implementing FROM-16 as part of a routine monitoring system could be preferable for tracking family impacts of ATMP treatment.

### Limitations and future research

As with any research, this study has several limitations that could be addressed through further research. We sought to validate the FROM-16 in the ATMP patient population and identify any domains of unique impacts felt by the family members. Sufficient participant numbers from both the questionnaires (*n* = 24) and interviews (*n* = 10) were achieved to demonstrate validity of the FROM-16 in this population and saturation was reached during the interviews. The misfit of the two-factor model confirmed by confirmatory factor analysis (CFA) in this study could have been largely due to the small sample size, which would have been expected for a study of this nature. Therefore, future work in this population should include repeating CFA analysis on a larger sample size, and further expand these findings through larger scale analysis. Secondly, the results of this study indicate that some participants gave minimised responses; this could potentially bias or skew results, This bias should be addressed in future studies, perhaps through conducting focus groups where participants may encourage each other to open up and discuss their difficulties in more detail through their shared experiences. Finally, this study solely investigated quality of life impact for family members of patients receiving ATMPs in England and Wales. Thus, the results of this research are context-specific, which could be alleviated through conducting further research in Scotland and Northern Ireland to develop a cohesive UK-based analysis. Extending this analysis to other countries, in Europen and beyond would also be fruitful.

## Conclusions

This study has confirmed that the FROM-16 is a suitable tool for assessing the quality-of-life impact on family members of patients receiving ATMPs. Using the FROM-16 as a part of a routine systematic monitoring of these family members may help to identify hidden impacts to their health.

Prospective completion of the FROM-16 before and after ATMP administration would quantify the impact of indicated diseases and conditions on the quality of life of family members and the improvement to this of treating the patient. This could provide additional evidence in future to strengthen the value-proposition for these high-cost treatments.

## Electronic supplementary material

Below is the link to the electronic supplementary material.

Supplementary Material 1
