# Supplementary material for: Validation of the FROM-16 in family members of patients receiving advanced therapy medicinal product (ATMP)
Source: Qual Life Res. 2025 Jan 25;34(4):949–62. doi: 10.1007/s11136-024-03880-0 (PMC11982087; doi:10.1007/s11136-024-03880-0)
Supplement: Supplementary file 1 — Supplementary Material 1 [file 11136_2024_3880_MOESM1_ESM.docx]

**Appendices**

Supplementary Table 1: Demographic characteristics of the study participants.

|  | **Patients** | **Family Members** |
| --- | --- | --- |
| **Total** | 24 | |
| **Sex** | | |
| **Male** | 12 | 8 |
| **Female** | 12 | 16 |
| **Age Bracket** | | |
| 0–17 | 2 | 0 |
| 18–29 | 2 | 0 |
| 30–39 | 0 | 3 |
| 40–49 | 4 | 3 |
| 50–59 | 3 | 3 |
| 60–69 | 5 | 10 |
| 70–79 | 8 | 5 |
| **Ethnic Group** | | |
| **White British** | 20 | 23 |
| **White Irish** | 1 | 0 |
| **Any Other White Background** | 1 | 0 |
| **White & Asian** | 1 | 0 |
| **Indian** | 1 | 1 |
| **Relationship** | | |
| **Married** | 18 | |
| **Non-married Partner** | 1 | |
| **Civil Partnership** | 1 | |
| **Parent** | 4 | |

Supplementary Table 2: Diagnoses and prescribed treatments

| **Treatment** | **No.** | **Diagnoses** |
| --- | --- | --- |
| **CAR-T^$^** | 9 | Diffuse Large B-Cell Lymphoma |
|  | 2 | Acute Lymphoblastic Leukaemia |
|  | 2 | Multiple Myeloma |
|  | 1 | Follicular Lymphoma |
|  | 1 | Mantle Cell lymphoma |
| **Monoclonal Antibodies** | 1 | Oesophageal Adenocarcinoma |
|  | 1 | Mesothelioma |
|  | 1 | Renal Cell Carcinoma |
|  | 1 | Stage III Hodgkin’s Lymphoma |
| **Mesenchymal Stromal Cells** | 3 | Primary Sclerosing Cholangitis |
| **Zolgensma** | 2 | Type 1 SMA^*^ |

^$^CAR-T: Chimeric Antigen Receptors Cell Therapy; ^*^SMA: Spinal Muscular Atrophy

Supplementary Table 3: Number of coding references associated with each FROM-16 item

| **Number** | **Item/Theme** | **Participants** | **References** |
| --- | --- | --- | --- |
| **All** | **All FROM-16 Themes** | 11 | 122 |
| **Domain 1** | **Emotional** | 8 | 23 |
| *1* | I feel worried | 6 | 14 |
| *2* | I feel angry | 1 | 2 |
| *2.1* | Angry | 1 | 1 |
| *2.2* | Not angry | 1 | 1 |
| *3* | I feel sad | 1 | 1 |
| *3.1* | Feeling sad | 0 | 0 |
| *3.2* | Not feeling sad | 1 | 1 |
| *4* | I feel frustrated | 2 | 3 |
| *5* | Difficult to talk to someone about thoughts | 0 | 0 |
| *6* | Caring for my family member is difficult | 3 | 3 |
| *6.1* | Is difficult | 2 | 2 |
| *6.2* | Not difficult | 1 | 1 |
| **Domain 2** | **Personal and Social** | **11** | **99** |
| *7* | Hard to find time for myself | 4 | 8 |
| *7.1* | Self-care | 2 | 3 |
| *8* | Everyday travel is affected | 4 | 4 |
| *9* | Eating habits are affected | 3 | 4 |
| *10* | Family activities are affected | 2 | 4 |
| *11* | Holidays | 9 | 20 |
| *11.1* | Booked holidays following treatment | 1 | 1 |
| *11.2* | Cannot travel due to illness | 8 | 9 |
| *11.3* | Limited options | 2 | 5 |
| *11.4* | Little impact | 2 | 2 |
| *12* | Sex life is affected | 3 | 6 |
| *13* | Work is affected | 8 | 22 |
| *13.1* | Back to work after treatment | 2 | 2 |
| *13.2* | No affect | 3 | 5 |
| *13.3* | Stop work | 2 | 4 |
| *13.4* | Work is limited or affected | 4 | 11 |
| *14* | Relationships with family members are affected | 10 | 23 |
| *14.1* | Child care | 2 | 3 |
| *14.2* | Positive | 2 | 5 |
| *15* | Increased expenses | 4 | 6 |
| *16* | Sleep is affected | 3 | 3 |
| *16.1* | Negative affect | 2 | 2 |
| **16.2** | Positive affect | 1 | 1 |
| **Code** | **Additional Codes** | **97** | **230** |
| *17* | Additional Emotional Impact | 5 | 8 |
| *17.1* | Anxiety | 2 | 3 |
| *17.2* | Stress | 3 | 3 |
| *18* | Cancelled life events | 2 | 2 |
| *19* | Changes to household routine | 5 | 19 |
| *19.1* | Additional responsibilities | 1 | 5 |
| *19.2* | Requiring additional equipment | 1 | 3 |
| *20* | COVID-19 | 7 | 30 |
| *21* | Finance | 6 | 14 |
| *21.1* | Reduced income | 4 | 8 |
| *22* | Help and support | 3 | 4 |
| *23* | Hospital appointments | 8 | 16 |
| *24* | Long-term planning | 3 | 11 |
| *25* | Patient health | 4 | 5 |
| *26* | Personal and couple activities | 8 | 21 |
| *27* | Personal health | 4 | 6 |
| *27.1* | Neglecting personal health | 1 | 2 |
| *28* | Social life | 6 | 22 |
| *28.1* | Negative Impact | 6 | 13 |
| *28.2* | Positive impact | 2 | 5 |
| *29* | Spontaneity | 3 | 5 |
| *30* | Treatment success | 7 | 11 |
| *31* | Treatment uncertainty | 2 | 5 |
| *32* | Variable impact | 1 | 6 |
| *33* | Changes to life after diagnosis | 3 | 3 |
